# Quantifying time perception during virtual reality gameplay using a multimodal biosensor-instrumented headset: a feasibility study

**DOI:** 10.3389/fnrgo.2023.1189179

**Published:** 2023-07-14

**Authors:** Marc-Antoine Moinnereau, Alcyr A. Oliveira, Tiago H. Falk

**Affiliations:** ^1^Institut National de la Recherche Scientifique (INRS-EMT), University of Québec, Montréal, QC, Canada; ^2^Graduate Program in Psychology and Health, Federal University of Health Sciences of Porto Alegre, Porto Alegre, Brazil

**Keywords:** physiological signals, virtual reality, features selection, remote experiment, machine learning

## Abstract

We have all experienced the sense of time slowing down when we are bored or speeding up when we are focused, engaged, or excited about a task. In virtual reality (VR), perception of time can be a key aspect related to flow, immersion, engagement, and ultimately, to overall quality of experience. While several studies have explored changes in time perception using questionnaires, limited studies have attempted to characterize them objectively. In this paper, we propose the use of a multimodal biosensor-embedded VR headset capable of measuring electroencephalography (EEG), electrooculography (EOG), electrocardiography (ECG), and head movement data while the user is immersed in a virtual environment. Eight gamers were recruited to play a commercial action game comprised of puzzle-solving tasks and first-person shooting and combat. After gameplay, ratings were given across multiple dimensions, including (1) the perception of time flowing differently than usual and (2) the gamers losing sense of time. Several features were extracted from the biosignals, ranked based on a two-step feature selection procedure, and then mapped to a predicted time perception rating using a Gaussian process regressor. Top features were found to come from the four signal modalities and the two regressors, one for each time perception scale, were shown to achieve results significantly better than chance. An in-depth analysis of the top features is presented with the hope that the insights can be used to inform the design of more engaging and immersive VR experiences.

## 1. Introduction

The use of virtual reality (VR) has increased significantly in recent years due to its numerous applications in various fields such as gaming, education, training, and therapy (Xie et al., 2021; Ding and Li, 2022). VR provides immersive and interactive environments that can simulate different scenarios and offer the user a unique experience. As new VR technologies emerge, the measurement of the quality of immersive experiences has become crucial (Perkis et al., 2020). For example, researchers and developers have become interested in understanding the psychological and physiological factors that influence the users' immersive quality of experiences, such as the sense of presence, immersion, engagement, and perception of time (Moinnereau et al., 2022a). While substantial work has been reported on presence, immersion, and engagement (e.g., Berkman and Akan, 2019; Muñoz et al., 2020; Rogers et al., 2020), very little work has been presented to date on characterizing the user's perception of time and the role it has on overall immersive media quality of experience.

Time perception is a complex cognitive process that allows individuals to estimate the duration and timing of events. It plays a crucial role in several aspects of human life, such as decision-making, memory, and attention (Wittmann and Paulus, 2008; Block and Gruber, 2014). Measuring a user's sense of perception of time in VR can be challenging due to potential confounds with sense of presence and flow (Mullen and Davidenko, 2021). Existing methods of measuring time perception in VR commonly rely on self-reports and questionnaires, such as the Metacognitive Questionnaire on Time (Lamotte et al., 2014). While these methods have provided valuable insights into the mechanisms of time perception, they have several limitations. For instance, they are prone to response biases and are influenced by various cognitive and contextual factors, such as attention and arousal (Askim and Knardahl, 2021). Furthermore, these methods may not capture the dynamic nature of time perception as they depend on the individual's ability to accurately perceive and report time. Moreover, questionnaires are often presented post-experiment, thus providing little insight for in-experiment environment adaptation to maximize the user experience.

Recently, there has been a push to use wearables and biosignals to measure, in real-time, cognitive and affective states of users while immersed in VR experiences (Moinnereau et al., 2022b,c,d). For example, electroencephalography (EEG) signals have been used to study engagement correlates within virtual reality experiences (Muñoz et al., 2020; Rogers et al., 2020). Electrocardiograms (ECG) have been used to uncover the relationship between valence and heart rate variability (HRV) (Maia and Furtado, 2019; Abril et al., 2020), while the research by Lopes et al. (2022) has established connections between relaxation, heart rate, and breathing patterns while the user is immersed in a virtual forest. Electrodermal activity (EDA), in turn, has been investigated to gain insights into the user's sense of presence and relaxation in virtual environments (Terkildsen and Makransky, 2019; Salgado et al., 2022). Furthermore, other studies have explored head movements to better comprehend users' reported levels of valence and arousal, as well as emotional states (Li et al., 2017; Xue et al., 2021). Eye movement has also been examined to assess factors such as immersion, concentration levels, and cybersickness in VR settings (Ju et al., 2019; Chang et al., 2021). The work by Moinnereau et al. (2022d) explored several neuro-psychological features as correlates of different user emotional states, engagement levels, and arousal-valence dimensions.

Despite all of these advances, limited research exists on the use of biosignals to monitor correlates of time perception while the user is immersed in VR. This is precisely the gap that the current study aims to address. In particular, two research questions are addressed: (1) what modalities and features provide the most important cues for time perception modeling and (2) how well can we objectively measure time perception. To achieve this goal, we build on the work from Moinnereau et al. (2022d) and show the importance of different modalities, including features extracted from EEG, eye gaze patterns derived from electro-oculography (EOG) signals, heart rate measures computed from ECG, and head movement information extracted from tri-axis accelerometer signals from the headset, for time perception monitoring. It is important to emphasize that given the exploratory nature of this study and the limited number of participants, our findings should be considered preliminary and indicative of the feasibility for objective time perception monitoring. Despite these limitations, this study contributes to the development of new methods to monitor time perception from biosignals, thus opening the door for future adaptive systems that maximize user experience on a per-user basis.

The remainder of this paper is organized as follows: Section 2 provides a review of the relevant literature in the field. Section 3 delves into the experimental procedures and details the bio-signal feature extraction pipelines utilized in this study. Section 4 presents the experimental results and contextualizes them within existing works. Finally, Section 5 offers concluding remarks.

## 2. Time perception research: background

Previous studies have investigated the connection between the brain and time perception, revealing that the frontal and parietal cortices, basal ganglia, cerebellum, and hippocampus are critical brain regions involved in the perception of time (Fontes et al., 2016). Specifically, the dorsolateral prefrontal right cortex has been identified as a significant region involved in time perception. EEG has been a common method used to study the neural correlates of temporal processing. EEG is a non-invasive technique that measures the electrical activity of the brain. It is important to note that time perception involves various psychological constructs, including attention, engagement, arousal, and even the influence of emotional stimuli. Each of these factors can modulate our perception of time in different ways, making time perception a multifaceted and dynamic process (Buhusi and Meck, 2005; Zakay, 2014). This complexity is reflected in the diverse range of methods and measures used to study time perception, as we will discuss in the following sections.

The work of Johari et al. (2023) examined semantic processing of time using EEG and found that the right parietal electrodes showed an event-related potential (ERP) response specific to the perception of event duration, with stronger alpha/beta band desynchronization. The work of Vallet et al. (2019) investigated the mechanisms involved in time perception related to emotional stimuli with equal valence and arousal levels using electrophysiological data. The results show that the ability to estimate time is influenced by various cognitive and emotional factors. Specifically, valence and arousal can modulate time perception, thus altering perceived duration. This highlights the importance of considering these factors when studying time perception, and the need for measures that can capture these dynamics.

In the work of Silva et al. (2022), in turn, the effects of oral bromazepam (a drug used for short-term treatment of anxiety by generating calming effects) on time perception were explored. The study monitored the EEG alpha asymmetry in electrodes associated with the frontal and motor cortex. The study found that bromazepam modulated the EEG alpha asymmetry in cortical areas during time judgment, with greater left hemispheric dominance during a time perception task. Moreover, the study of Ghaderi et al. (2018) explored the use of EEG absolute power and coherence as neural correlates of time perception. They found that participants who overestimated time exhibited lower activity in the beta band (18–30 Hz) at several electrode sites. The study suggested that although beta amplitude in central regions is important for timing mechanisms, its role may be more complex than previously thought. Lastly, the work by Kononowicz and van Rijn (2015) investigated correlates of time perception and reported on the importance of beta and theta frequency subbands.

Moreover, eye movements have become a valuable tool to investigate temporal processing and its relation to attention and eye gaze dynamics. Recent studies, for example, have highlighted the close link between eye movement and time perception, revealing that time compression could be due to the lack of catch-up saccades (Huang et al., 2022). Other works have linked saccade and microsaccade misperceptions (Yu et al., 2017), as well as their role in visual attention and, consequently, on time perception (Cheng and Penney, 2015). Finally, it has been reported that when short intervals between two successive perisaccadic visual stimuli are underestimated, a compression of time is perceived (Morrone et al., 2005). These findings emphasize the importance of eye movements in understanding temporal processing, its connection to visual perception, and how our perception of time is influenced not only by our cognitive and emotional state but also by our visual attention and gaze dynamics.

Physiological measures, such as heart rate variability (HRV) and skin conductance, have also been used to explore the mechanisms underlying time perception. A study found that low-frequency components of HRV were associated with less accurate time perception, suggesting that the autonomic nervous system function may play a crucial role in temporal processing (Fung et al., 2017). Another study showed that increased HRV was linked to higher temporal accuracy (Cellini et al., 2015). Additionally, changes in the sympathetic nervous system (SNS) activity have been found to affect time perception, with research showing that increased SNS activity, indicated by elevated heart rate and frequency of phasic skin conductance response, was linked to the perception of time-passing more quickly (Ogden et al., 2022). These findings highlight the importance of physiological measures in understanding the complex interaction between the body and time perception.

As can be seen, while numerous studies exist exploring the use of psychophysiological measures to characterize the perception of time, their measurement in virtual reality has remained a relatively unexplored area of research. Perception of time in VR has relied mostly on participant-provided ratings of judgement of time, usually provided post-experiment (Volante et al., 2018). Being able to track time perception objectively in real time could be extremely useful for immersive experiences. While time compression has been linked with high levels of engagement and attentional resources (Read et al., 2022), time elongation could also be linked to boredom (Igarzábal et al., 2021). As such, time perception monitoring could be extremely useful for user experience assessment. In the next section, the materials and method used to achieve this goal are described.

## 3. Materials and methods

In this section, we detail the experimental protocol followed, including the dataset used, signal pre-processing, feature extraction and selection methods, and the regression method used.

### 3.1. Experimental procedure and time perception ratings

**Figure 1:**
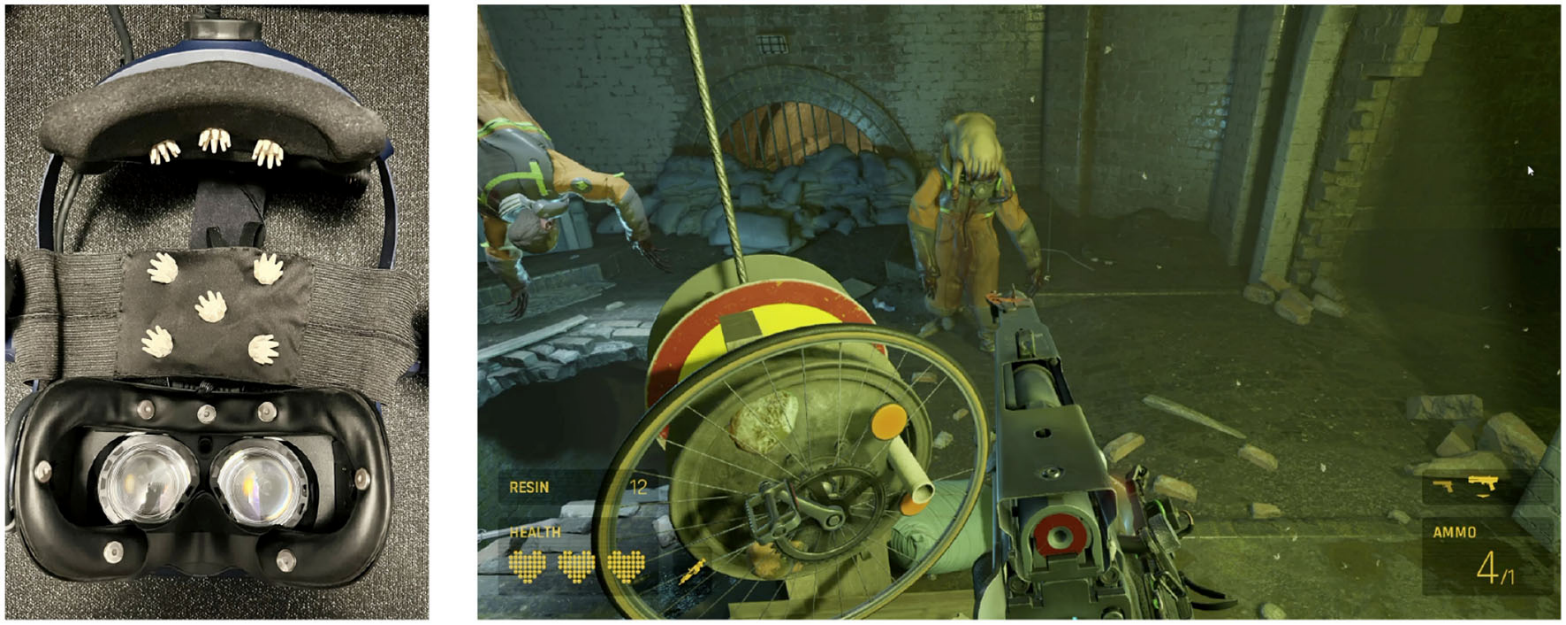
**(Left)** 16 ExG biosensor-instrumented VR headset used to collect data; **(Right)** Participant's view of a scene played in the VR game Half-Life: Alyx.

The experimental protocol followed in this study was designed to ensure the collection of high-quality data while minimizing experimenter intervention. The dataset used in this study has been described in detail by Moinnereau et al. (2022d). Here, we provide a comprehensive summary of the data and the experimental procedure and the interested reader is referred to Moinnereau et al. (2022d) for more details. An instrumented headset was created following advice by Cassani et al. (2020); Figure 1 (left) shows the instrumented HTC VIVE Pro Eye headset equipped with 16 ExG sensors. All data were recorded using the OpenBCI system (OpenBCI, United States) with a sampling rate of 125 Hz, ensuring synchronization across all signals. Data was collected during the COVID-19 lockdown. The instrumented headset along with the necessary accessories (e.g., laptops, controllers) was dropped off at participants' homes and later picked up and sanitized following protocols approved by the authors' institution. Eight participants (five male and three female, 28.9 ± 2.9 years of age), all of whom were students at the authors' institution, consented to participate in the experiment, which involved playing the VR game Half-Life Alyx, as shown in Figure 1 (right). The game is a first-person shooter game combining elements of exploration, puzzle-solving, combat, and story. During the action parts, players need to get supplies, use interfaces, throw objects, and engage in combat.

Participants were given instructions remotely (via videoconference) on how to place the headset and minimal experimenter intervention was needed. The experimenter would show participants how to assess signal quality utilizing in-house developed software. When signal quality was deemed acceptable, participants could play the game at their own pace and the videoconference call was terminated. In the experiment, participants engaged in two distinct tasks, referred to as “baseline” and “fighting/shooting.” The “baseline” task corresponds to the initial two chapters of the game (~30 min of gameplay), during which the player becomes familiar with the game storyline, learns navigation techniques, and practices manipulating in-game objects. The “fighting/shooting” task, which follows the “baseline” task, involves more complex gameplay, including puzzle-solving and combat challenges (~1 h of gameplay). Participants played on average a total of 1.5 h, resulting in close to 10 h of physiological data recordings. At the end of each condition, participants responded to the unified experience questionnaire that combines items compiled from 10 different scales, including sense of presence, engagement, immersion, flow, usability, skill, emotion, cybersickness, judgement, and technology adoption, all using a 10-point Likert scale (Tcha-Tokey et al., 2016). The work by Moinnereau et al. (2022d) compared the changes reported by the participants between the baseline and the fighting/shooting tasks. Here, we combine both tasks and explore the use of the biosignals in monitoring the two ratings provided to two questions related to time perception: Q1—“*Time seemed to flow differently than usual”* and Q2—“*I lost the sense of time”*.

### 3.2. Feature extraction

**Figure 2:**
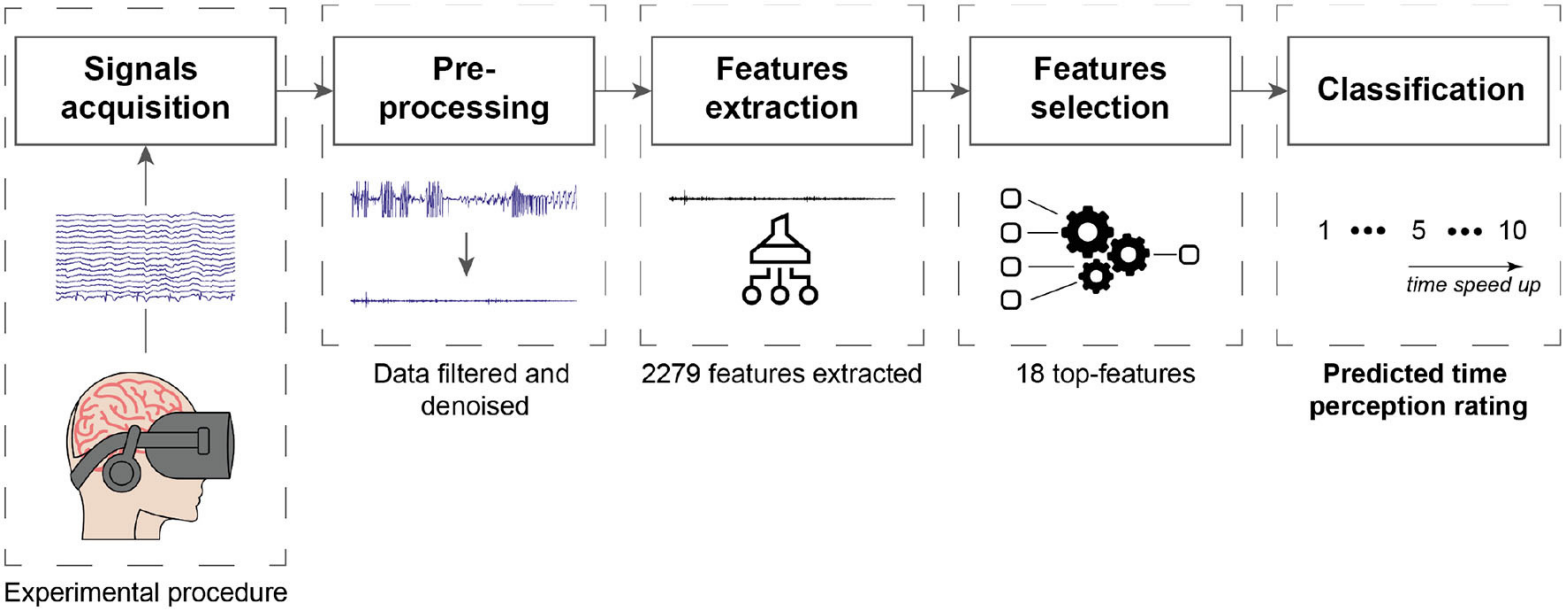
Processing pipeline to predict time perception ratings from biosignals.

To answer our first research question, we extracted several features from the EEG, EOG, ECG, and accelerometer data. These features were selected based on their potential relevance to time perception and cognitive processing in VR. For a comprehensive understanding of our methodology, including feature extraction and subsequent steps, please refer to the processing pipeline illustrated in Figure 2. In the following sections, we provide a detailed description of the different features we used and how they were extracted from the physiological signals. All of these features are summarized in Table 1.

**Table 1:** Computed features to predict time perception.

**Modality**	**Features group**	**Features**
EEG	Frequency band and ratios (computed on electrodes average)	delta, theta, alpha, beta, gamma
		delta/theta, delta/alpha, delta/beta, delta/gamma
		theta/delta, theta/alpha, theta/beta, theta/gamma
		alpha/delta, alpha/theta, alpha/beta, alpha/gamma,
		beta/delta, beta/theta, beta/alpha, beta/gamma,
		gamma/delta, gamma/theta, gamma/alpha, gamma/beta
	Metrics (skewness and kurtosis computed for each metric)	ES
		AI
		VI
		FAA
	Magnitude square coherence (computed for each of the 55 pairs)	msc-delta
		msc_theta
		msc_alpha
		msc_beta
		msc_gamma
	Phase coherence (computed for each of the 55 pairs)	phc-delta
		phc-theta
		phc-alpha
		phc-beta
		phc-gamma
	Amplitude modulation rate-of-change (computed for each of the 55 pairs)	delta-mdelta
		theta-mdelta
		theta-mtheta
		alpha-mdelta
		alpha-mtheta
		beta-mdelta
		beta-mtheta
		beta-malpha
		beta-mbeta
		gamma-mdelta
		gamma-mtheta
		gamma-malpha
		gamma-mbeta
		gamma-mgamma
EOG	Eye blink and saccades	blink duration, blink count, saccades duration, saccades count
	Time-domain	mean, standard deviation, peak value
	Frequency-domain	mean frequency, median frequency
	Statistical	interquartile range, variance, energy
	Eye movement	total number of up-down and left-right shifts
ECG	Time-domain	HR, IBI mean, SDNN, RMSSD, NN50, pNN50
	Frequency-domain	LF, HF, ULF, VLF
	Nonlinear-domain	SD1, SD2
ACC	Statistics along *x, y*, and *z* axes (computed for acceleration, velocity and displacement)	mean, standard deviation, skewness, kurtosis, energy

#### 3.2.1. EEG features

In this study, the total 1.5 h of data collected per subject was divided into 5-min window. To prepare the EEG signals for feature extraction, we first applied a finite impulse response band-pass filter with a range of 0.5–45 Hz using the EEGLab toolbox in Matlab. Next, we utilized the artifact subspace reconstruction (ASR) algorithm, from the EEGLAB plugin with default parameters, to enhance the signal quality and remove motion artifacts (Mullen et al., 2015). These parameters were shown in our previous work (Moinnereau et al., 2022d) to accurately remove artifacts from the EEG signals. This pre-processing step ensured that the EEG signals were of high quality and suitable for feature extraction. Before the beginning of each game session, it is important to note that the initial loading time of the game was utilized as a reference point for calibrating the ASR algorithm for each participant. Lastly, each 5-min window was further segmented into 2-s epochs with a 50% overlap for features extraction.

##### 3.2.1.1. Power spectral features, ratios and metrics

Next, the power distribution of the five EEG frequency bands was computed: delta (1–4 Hz), theta (4–8 Hz), alpha (8–12 Hz), beta (12–30 Hz), and gamma (30–45 Hz) using the Welch method. For each frequency band, the average power was calculated across all EEG channels, and four ratios were computed for each of the average power of each frequency band, resulting in 20 features. Indeed, previous research has shown that certain EEG band ratios, such as delta-beta coupled oscillations, play a crucial role in temporal processing (Arnal et al., 2014). These features provided valuable information on the power distribution of different frequency bands in the EEG signals, which are known to be associated with various cognitive and emotional states.

In addition to the EEG band ratio features, we extracted four indexes to further explore the participants' mental states during the task, namely engagement, arousal, valence, and frontal alpha asymmetry. These measures have been shown to be related to cognitive and emotional processes that are involved in time perception, such as attention, motivation, and affective valence (Yoo and Lee, 2015; Polti et al., 2018; Read et al., 2021). The engagement score (ES), for instance, is a measure of the participant's attention and involvement in the task, which can directly influence their perception of time. Similarly, arousal and valence indexes (AI and VI respectively) provide insights into the participant's emotional state during the task, which has been shown to affect time perception. The frontal alpha asymmetry index (FAA), on the other hand, is a measure of the balance of power between the left and right frontal cortex, which has been linked to emotional valence and motivation, both of which can influence time perception.

**Figure 3:**
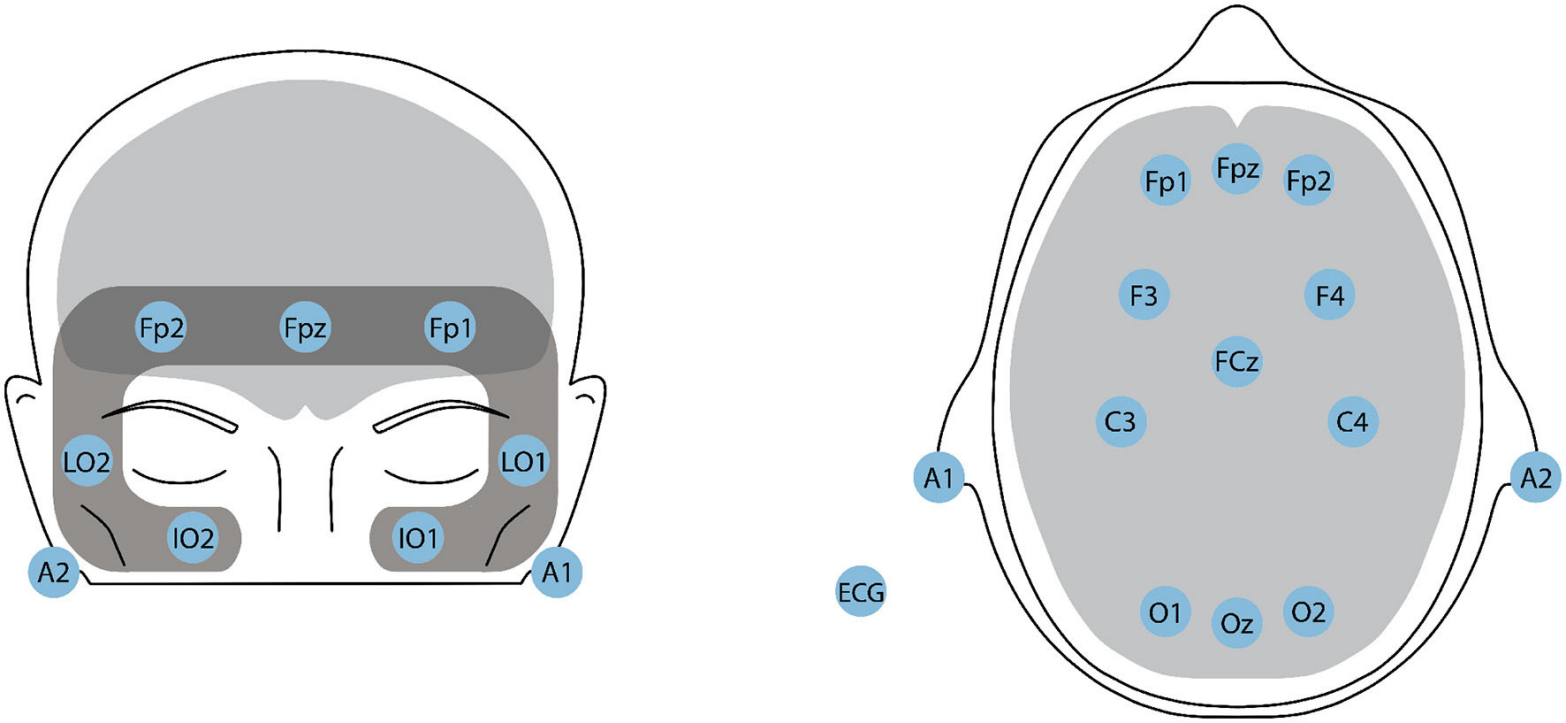
Headplots showcasing the positioning of the 16-ExG electrodes on the HTC VIVE Pro Eye VR headset. **(Left)** The 7-channel EOG montage, with LO1 and LO2 for horizontal EOG (left and right cantus respectively), and Fp1, Fpz, Fp2 (above superior orbit), IO1, and IO2 (below inferior orbit) for vertical EOG. **(Right)** The 11-channel EEG montage, with electrodes placed in the prefrontal, frontal, central, and occipital areas. A1 and A2 serve as references. An additional sensor is placed on the left collarbone for ECG recording.

To calculate ES, we first computed the relative powers by summing the absolute power across the delta, theta, and alpha bands, dividing each individual band's absolute power by the total power, and expressing it as a percentage in the Fp1 channel. AI and VI were calculated using the F3 and F4 channels. Finally, FAA was calculated by subtracting the log-power of the alpha EEG band in electrode F4 from the log-power of the alpha band from electrode F3. The electrode locations are illustrated in Figure 3. Additionally, the skewness and kurtosis of each of these four indexes were also calculated, resulting in a total of 12 additional features for EEG modality. More details about these features can be found in Moinnereau et al. (2022d).

##### 3.2.1.2. Phase and magnitude spectral coherence

To measure the connectivity between cortical regions, we used the Phase and Magnitude Spectral Coherence (PMSC) features, represented in Table 1 by *phc*-*band* and *msc*-*band* for phase coherence and magnitude spectral coherence respectively. These features measure the co-variance of the phase and magnitude between two signals. The motivation for the inclusion of connectivity measures as possible features is based on the hypothesis that the level of connectivity could potentially indicate changes in time perception within the immersive virtual environment (Lewis and Miall, 2003). In our case, with 15 electrodes, we computed PMSC for all possible pairs of electrodes for each of the five sub-bands (δ, θ, α, β, γ). Given that there are 55 unique electrode pairs and 5 sub-bands, this results in a total of 550 PMSC features. For the computation of PMSC, including the complex coherency function and cross-spectral densities, we followed the method detailed in Aoki et al. (1999). By using PMSC, we were able to examine the functional connectivity between different brain regions, which could provide insights into how individuals perceive time in virtual environments. This could potentially help identify the neural mechanisms underlying accurate time perception in immersive virtual experiences.

##### 3.2.1.3. Phase and magnitude spectral coherence of amplitude modulation features

Moreover, we utilized the Phase and Magnitude Spectral Coherence of Amplitude Modulation (PMSC-AM) features, which extend the capacity of PMSC features to amplitude modulations and provide information about the rate-of-change of specific frequency sub-bands. These interactions are represented by the notation *band*_*mband* and reveal interactions between different brain processes and long-range communication. These features have proven to be useful in predicting arousal and valence by Clerico et al. (2018). Given the established relationship between arousal and valence and time perception (Yoo and Lee, 2015; Jeong-Won et al., 2021), here we explore the potential use of these features as correlates of time perception. The PMSC-AM features were calculated based on the modulated signals of each band, resulting in a total of fifteen signals per channel. The magnitude spectral coherence and phase coherence were then computed for all channel pairs, resulting in a total of 1,540 features. More details about the PMSC and PMSC-AM features can be found in Clerico et al. (2018).

#### 3.2.2. EOG features

The EOG signal has a frequency range of 0.1–50 Hz and amplitude between 100 and 3,500 μV (López et al., 2017). In this study, we extracted eye blink and saccades measures from the 5-min windows using the EOG event recognizer toolbox in Matlab (Toivanen et al., 2015), providing information on blink duration, blink count, saccade duration, and saccade count. These metrics have been shown to have a correlation with time perception (Morrone et al., 2005; Huang et al., 2022).

Next, we extracted time-domain and frequency-domain features from the EOG signals using the signal processing toolbox in MATLAB. These features were extracted from 2-s epochs of the EOG signals, with a 50% overlap between consecutive intervals. The time-domain features include measures such as mean, standard deviation, skewness, and kurtosis, while the frequency-domain measures include mean frequency, median frequency, peak amplitude, and frequency location of the peak amplitude. These features provide information on the distribution of power across different frequency bands in the EOG signals and can reveal patterns related to eye movement. We also calculated statistical features such as interquartile range, variance, and energy. In total, we extracted 31 features from the EOG signals, providing valuable information on eye movements during gameplay.

In addition to the previously mentioned EOG features, we also extracted eye movement features related to the number of times eye movements shifted from the upper to the lower quadrant, as well as from the left to the right quadrant in the field of view of the VR headset. To this end, we employed a Support Vector Machine (SVM) classifier, as proposed by Moinnereau et al. (2020). This classifier was trained on a separate dataset where eye direction was tracked across 36 distinct points within the visual field, each separated by an angle of 10 degrees. For each 500 ms window within the seven EOG signals recorded by the instrumented headset from electrode locations Fp1, Fpz, Fp2, horizontal EOG right, horizontal EOG left, vertical EOG right, and vertical EOG left (namely, the sensors placed on the faceplate of the headset, as shown in Figure 1), we calculated the signal slope and input it into the SVM classifiers. These classifiers were designed to discern between up-down and left-right eye movements. The output of the classifier provided the eye's direction, and we subsequently calculated the number of gaze shifts between the quadrants of interest. These shifts were then incorporated as two additional features within our EOG feature set.

#### 3.2.3. ECG features

Both heart rate (HR) and HRV have been linked to time perception, with studies suggesting that fluctuations in these physiological markers can influence the experience of time (Meissner and Wittmann, 2011; Pollatos et al., 2014). Therefore, we extracted ECG features from the 5-min windows using an open-source MATLAB toolbox to gather 15 features that relate to HR and HRV.^1^ The analysis of HRV can be categorized into three methods: time-domain, frequency-domain, and nonlinear methods. The time-domain features measured the variation in time between two successive heartbeats, or interbeat intervals (IBI). We extracted the average IBI, the standard deviation of NN intervals (SDNN), the root mean square of successive RR interval differences (RMSSD), the number of pairs of successive RR intervals that differ by more than 50 ms, and the percentage of this difference (NN50 and pNN50, respectively). Frequency-domain analysis focuses on the power spectral density of the RR time series. We obtained the relative power of the low-frequency (LF) band (0.04–0.15 Hz) and high-frequency (HF) band (0.15–0.4 Hz), as well as their percentages. We also calculated the ratio of LF to HF and the total power, which is the sum of the four spectral bands, LF, HF, the absolute power of the ultra-low-frequency (ULF) band ( ≤ 0.0003 Hz), and the absolute power of the very-low-frequency (VLF) band (0.0033–0.04 Hz). Finally, nonlinear measurements were used to assess the unpredictability of a time series. We extracted the Pointcare plot standard deviation perpendicular to the line of identity (SD1) and along the line of identity (SD2). These features provide valuable insights into the variability of HR and HRV during the VR gameplay session.

#### 3.2.4. Accelerometer features

The OpenBCI bioamplifier includes an accelerometer, which was placed toward the back of the VR headset. This allows for the analysis of head motion and orientation, which is crucial as head movements have been associated with influencing temporal perception and varying based on arousal and valence states (Behnke et al., 2021). In fact, the speed and nature of head movements can affect the perception of simultaneity between sensory events (Sachgau et al., 2018; Allingham et al., 2020) and can lead to recalibration of time perception in a virtual reality context (Bansal et al., 2019). To capture these dynamics, we extracted features from the *x, y*, and *z* signals of the accelerometer data, segmented into 2-s epochs with a 50% overlap. Specifically, we extracted statistical measures (i.e., mean, standard deviation, skewness, kurtosis, and energy) for acceleration, velocity, and displacement along the *x, y*, and *z* axes. This resulted in a total of 117 features that provide valuable information about the user's head movements during the VR experience.

### 3.3. Feature selection

Feature selection is an important step in classification tasks that involves the removal of irrelevant or redundant features, thus providing dimensionality reduction prior to classification. In our study, given the relatively small amount of data collected, feature selection is particularly important. We performed a two-step process for feature selection using built-in functions in Matlab: first, we applied the Spearman correlation coefficient to identify features with a medium to high correlation with the ratings from Q1 and Q2; second, we applied the minimum redundancy maximum relevance (mRMR) algorithm to these selected features. The Spearman correlation coefficient measures the strength and direction of the relationship between the physiological features extracted and the ratings of the two questions on time perception. Specifically, we performed a Spearman correlation between all the 2,279 features extracted from each 5-min window of the entire dataset, which includes all recordings from all participants, and the ratings of the two questions Q1 and Q2. To align the number of ratings with the number of 5-min window, we replicated the same rating for each window within a single recording. This approach allowed us to assess the relationship between each feature and the time perception ratings across all 5-min windows and all participants. Spearman correlation is a non-parametric measure that assesses the monotonic relationship between two variables (Schober et al., 2018); hence, does not assume linearity, making it suitable for our analysis. Only features with a Spearman correlation coefficient >0.3 or < −0.3 were retained, indicating a medium to high correlation.

Next, we applied the minimum redundancy maximum relevance (mRMR) algorithm (Peng et al., 2005) on the physiological features that showed significant Spearman correlations with the ratings from Q1 and Q2. The mRMR method finds the most relevant features for the classification task and removes features with high mutual information to minimize redundancy. This approach helps to reduce the dimensionality of the data, improves classification performance, and avoids overfitting. This selection method has been show to be very useful for biosignal data (Clerico et al., 2018; Jesus et al., 2021; Rosanne et al., 2021). As our dataset is small, we used five-fold cross-validation in our analysis. In this process, the dataset was divided into five subsets. For each fold, 80% of the data was used to calculate the mRMR, and the top features were recorded. This process was repeated five times, each time with a different subset held out. At the end, we compare the features that were consistently present across the five folds and use these as candidate features for time perception monitoring. With this analysis, a total of 18 top-features were found to be present in at least two of the five folds.

### 3.4. Regression, testing setup, and figures-of-merit

With the top-18 ranked features found, we employed a Gaussian process regressor (GPR) with a rational quadratic kernel to predict the two time perception ratings (Williams and Rasmussen, 1995) with the aim of answering our research question #2. This process was implemented using the Regression Learner toolbox in MATLAB. To find the optimal number of features to use, top-ranked features were added one by one and three figures-of-merit were used, namely root mean square error (RMSE), mean absolute error (MAE), and the *R*-squared (*R*^2^). Both RMSE and MAE provide insights into to overall error distribution of the predictor, with RMSE providing greater emphasis to larger errors. In both cases, lower values are better. The *R*-squared measure, in turn, measures the goodness of fit of the data to the regression model; higher values are desired. These three figures-of-merit are widely used in regression to assess the performance of the model.

For the analysis, a bootstrap testing methodology was followed where the data was randomly partitioned into 80% for training and 20% for testing and this partitioning was repeated 100 times. Lastly, to gauge if the obtained results were significantly better than chance, a “random regressor” was used. With this regressor, the same bootstrap testing setup was used, but instead of training the regressor with the true ratings reported by the participants, random ratings between 1 and 10 were assigned. To test for significance, a Kruskal–Wallis test was used (Kruskal and Wallis, 1952) for each of the metrics (RMSE, MAE, and *R*^2^).

## 4. Experimental results

**Figure 4:**
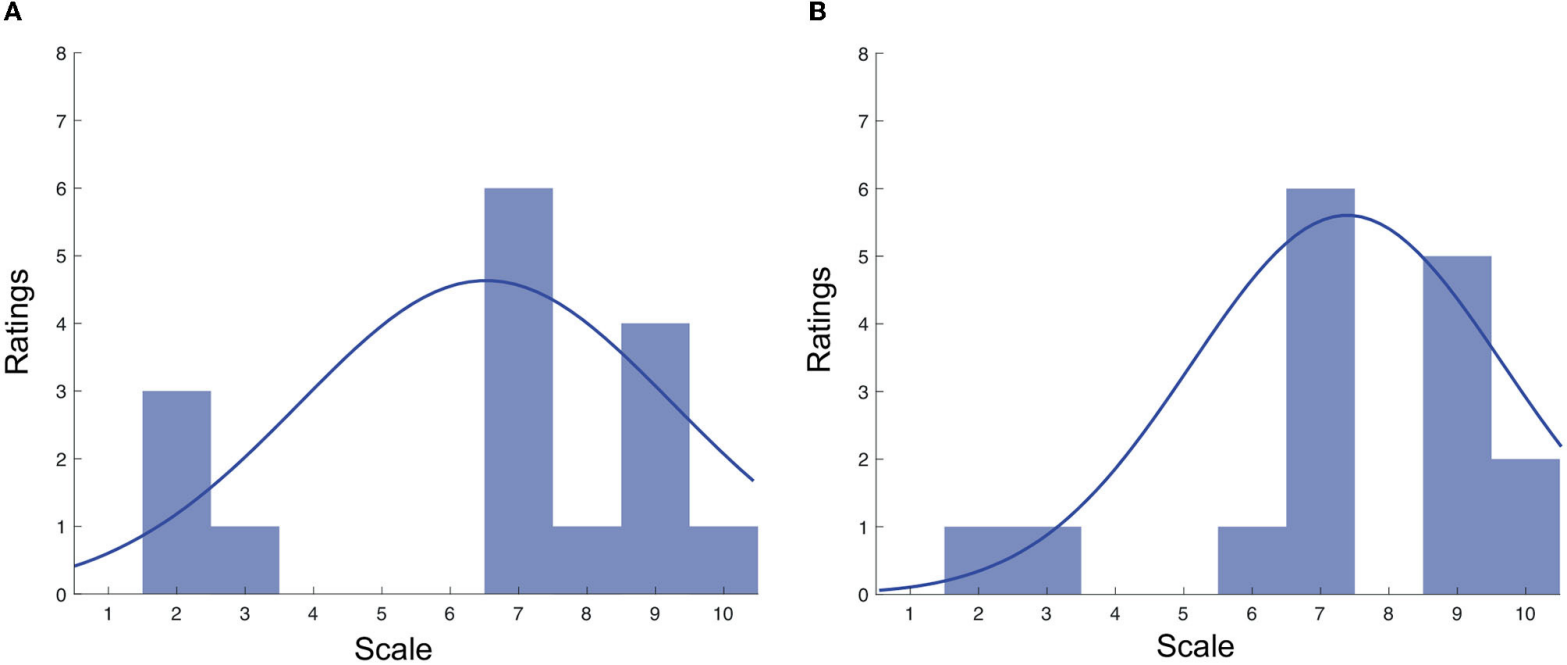
**(A)** Distribution of Q1 ratings across both testing conditions; **(B)** Distribution of Q2 ratings across both testing conditions.

Figures 4A, B display the distribution of the Q1 and Q2 ratings, respectively. For Q1, the ratings ranged from 2 to 10, with the majority being in the range of 7–9. The mean rating for Q1 was 6.40 (SD = 2.78). Similar findings were seen for Q2, where an average rating of 7.37 (SD = 2.27) was seen.

**Figure 5:**
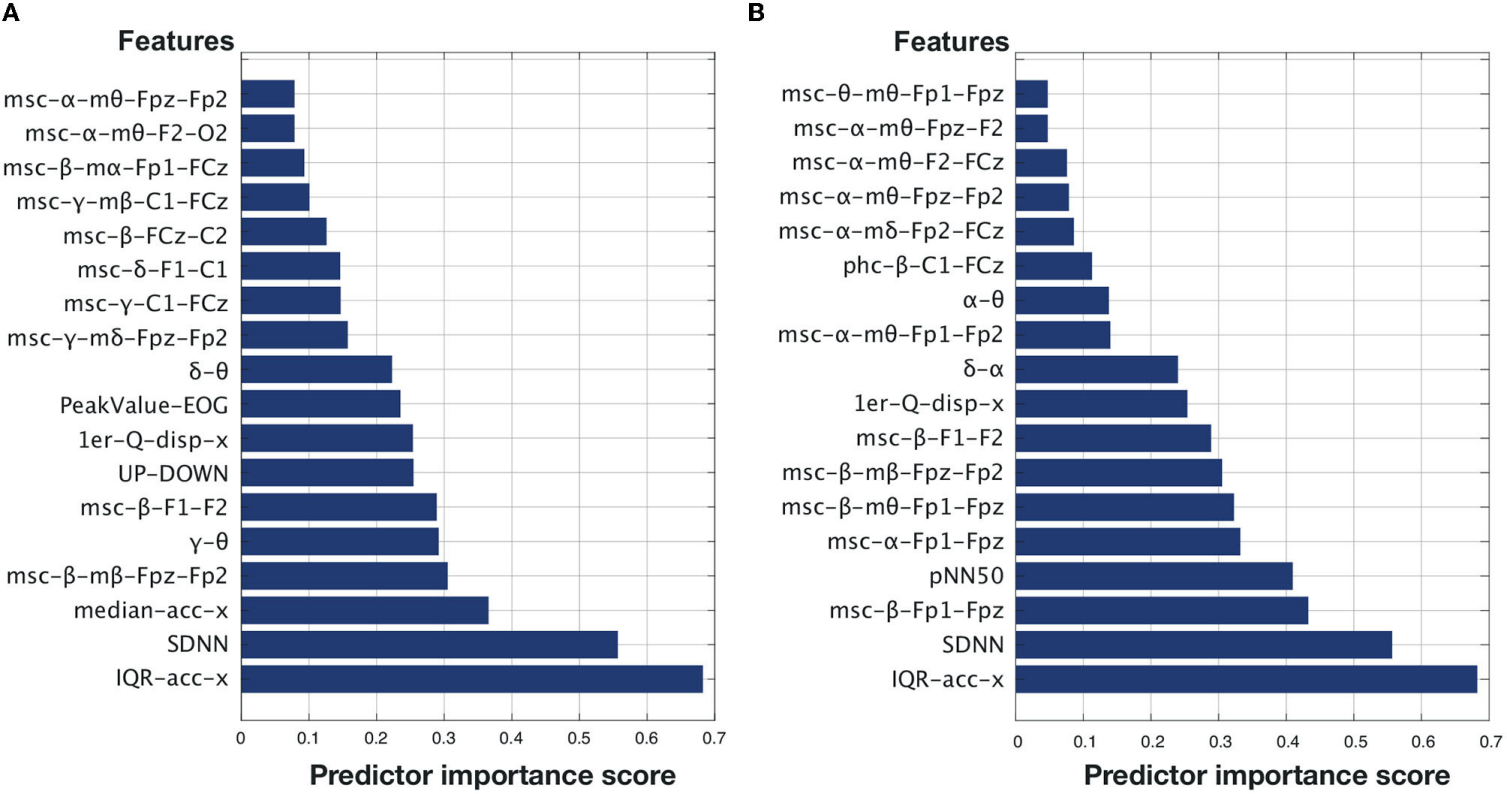
**(A)** Top features ranked for Q1; **(B)** Top features ranked for Q2.

A total of 2,279 features were extracted from the EEG, EOG, ECG, and accelerometer signals. After passing through the two feature ranking steps mentioned in Section 3.3, 18 top features were found for each of the two time perception ratings. Figures 5A, B display the selected features for Q1 and Q2, respectively, arranged in ascending order of importance given by the mRMR selection algorithm. As can be seen, for both Q1 (“*Time seemed to flow differently than usual”*) and Q2 (“*I lost the sense of time”*), head movement and HRV-related measures corresponded to the top-two most important features. The majority of the other top features are related to EEG measures of coherence between different electrode sites. For Q1, two EOG measures stood out, one of them corresponding to a number of shifts from the top-bottom quadrants based on outputs from the EOG-based classifier described by Moinnereau et al. (2020).

Table 2 reports the impact that including these top features one-by-one has on regressor accuracy. The goal of this analysis is to explore the optimal number of features for time perception monitoring. As can be seen from the Table, there is an elbow point for Q1 at seven features and at eight features for Q2. In fact, for Q2, the accuracy achieved with just two features was very close to that achieved with 8. For Q1, the top-seven features included: IQR-acc-x, SDNN, median-acc-x, msc-beta-mbeta-Fpz-Fp2, gamma-theta ratio, msc-beta-F1-F2, and UP-DOWN. For Q2, the top eight features correspond to IQR-acc-x, SDNN, pNN50, msc-beta-Fp1-Fpz, msc-alpha-Fp1-Fpz, msc-beta-mtheta-Fp1-Fpz, msc-beta-mbeta-Fpz-Fp2, msc-beta-F1-F2.

**Table 2:** Figures-of-merit as a function of number of features used to train the regressor for Q1 and Q2 ratings.

	**Q1**			**Q2**		
**Nb of features**	**RMSE**	**MAE**	*R* ^2^	**RMSE**	**MAE**	*R* ^2^
1	1.82	1.36	0.60	1.39	0.97	0.64
2	1.55	1.19	0.71	1.13	0.77	0.76
3	1.56	1.22	0.71	1.45	1.02	0.60
4	1.43	1.11	0.75	1.45	0.92	0.60
5	1.33	1.01	0.79	1.40	0.95	0.63
6	1.07	0.85	0.86	1.30	0.93	0.68
7	0.95	0.76	0.89	1.37	0.92	0.64
8	1.09	0.86	0.86	1.13	0.76	0.76
9	1.12	0.88	0.85	1.14	0.78	0.75
10	1.03	0.80	0.87	1.15	0.80	0.75
11	1.20	0.95	0.82	1.52	0.92	0.56
12	1.30	1.02	0.80	1.50	0.89	0.57
13	1.33	1.04	0.79	1.51	0.89	0.57
14	1.42	1.10	0.75	1.53	0.91	0.55
15	1.65	1.22	0.67	1.61	1.03	0.51
16	1.57	1.20	0.70	1.60	1.01	0.51
17	1.53	1.16	0.72	1.63	1.01	0.50
18	1.56	1.18	0.71	1.68	1.04	0.46

**Figure 6:**
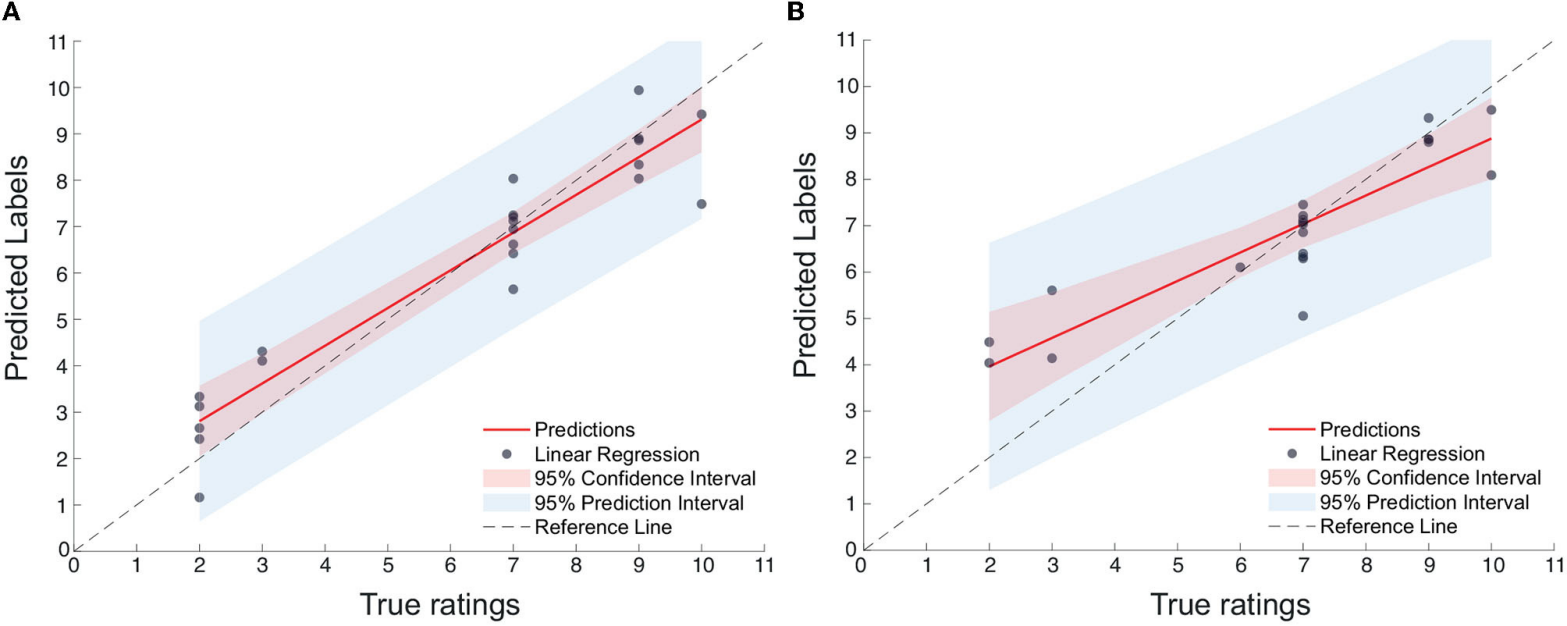
**(A)** Scatterplot of predicted vs. true ratings for Q1 (number of features = 7); **(B)** Scatterplot of predicted vs. true ratings for Q2 (Number of features = 8).

**Figure 7:**
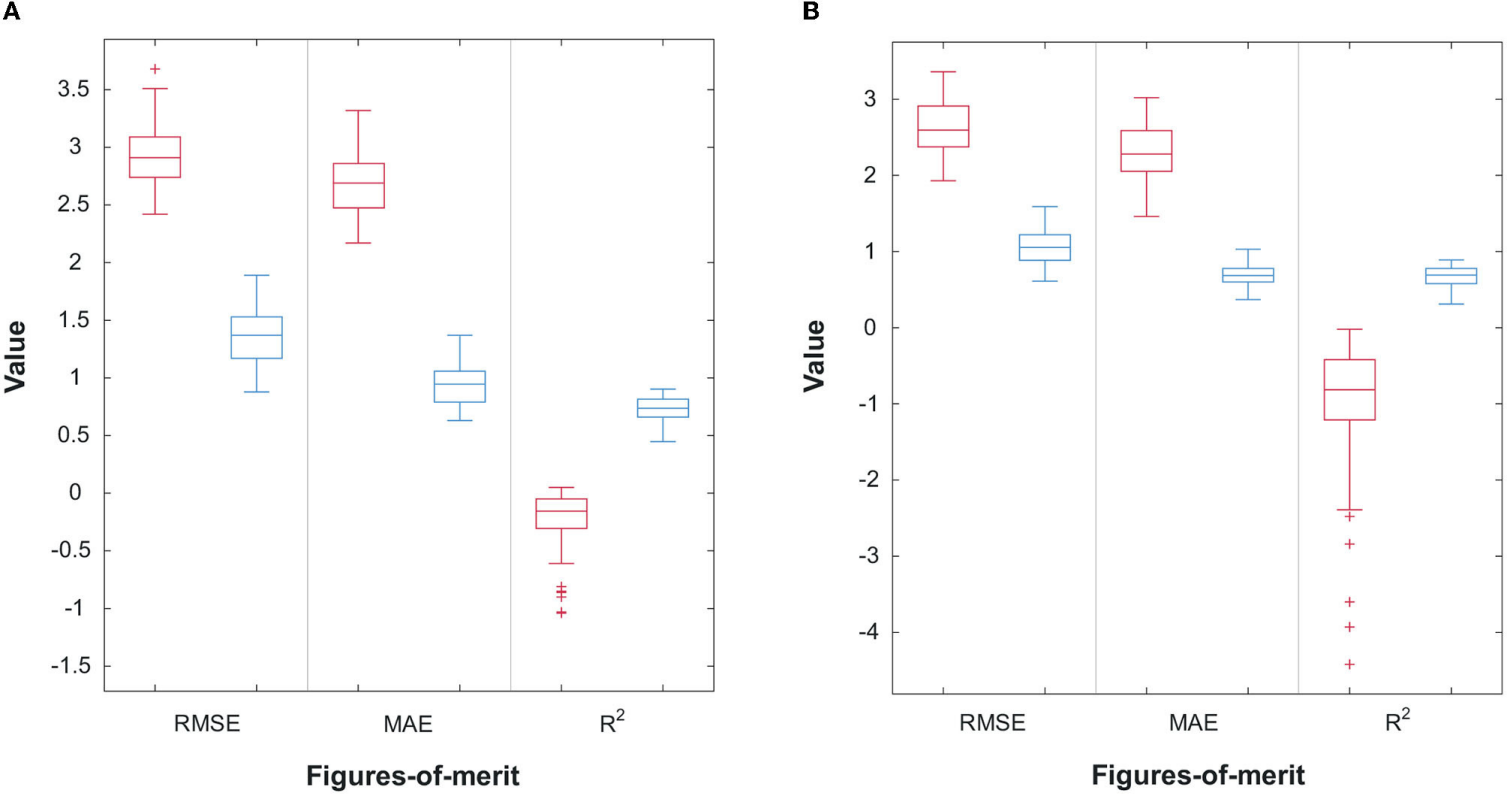
**(A)** Performance comparison of 100 random bootstrap trials for the random (red) and proposed (blue) regressors for Q1; **(B)** Performance comparison of 100 random bootstrap trials for the random (red) and proposed (blue) regressors for Q2.

Figures 6A, B display the scatterplots, including confidence intervals, of predicted vs. true subjective ratings for Q1 and Q2, respectively, using the top-7 and top-8 features mentioned above for one of the bootstrap runs. The reference, perfect-correlation line is included for comparisons. For Q1, a significant correlation of 0.95 can be seen between predicted and true ratings. For Q2, in turn, a significant correlation of 0.90 is observed. To test if these results are significantly better than chance, the prediction task is repeated 100 times using a bootstrap method. Figures 7A, B show the boxplots of the figures-of-merit achieved with the chance regressor and the proposed regressors for 100 bootstrap runs. As can be seen, the error based measures from the chance regressor result in similar trends and are almost three times as higher as the proposed method. Lastly, the Kruskal–Wallis test was performed over the entire 100 bootstrap trials and showed a significant difference (*p*-value = 10^−34^) for all three figures-of-merit.

## 5. Discussion

### 5.1. Time perception ratings

Our findings suggest that the majority of the participants experienced altered time perception during the experiment, with the majority strongly agreeing that time seemed to flow differently than usual for them and that they lost the sense of time while in gameplay. This is consistent with previous research on time perception in VR, which has demonstrated that the sense of time passing can be significantly influenced by the level of immersion and engagement with the virtual environment (Mullen and Davidenko, 2021; Read et al., 2022). The VR environment used in our study was a highly-rated videogame known for its immersive qualities. This emphasizes the importance of examining the relationship between immersion and time perception in VR settings.

To empirically investigate this relationship, we conducted a correlation analysis between the Q1 ratings and the ratings from the seven items in the immersion scale. A significant correlation of 0.81 was found between the Q1 ratings and the ratings from the seven items in the immersion scale, particularly with the item—“*I felt physically fit in the virtual environment”*. This strong correlation suggests that the more participants felt physically fit and comfortable in the virtual environment, the more they experienced altered time perception. This provides empirical support for the link between immersion and time perception, reinforcing the idea that immersion can significantly influence how individuals perceive time in VR. Interestingly, lower values were observed during the puzzle-solving tasks, as shown in Figure 4, which were rated by the participants as being less engaging than the shooting tasks. This further corroborates the link between engagement, immersion, and altered time perception in VR. The immersive qualities of the VR environment, combined with the engaging nature of the tasks, appear to have a significant impact on participants' perception of time.

### 5.2. Feature importance

The identified features provide valuable insights into the neural, physiological, and behavioral correlates of time perception in VR. The prominence of head movement and HRV-related measures among the top features for both Q1 and Q2 underscores the integral role of physical engagement and physiological responses in shaping time perception in VR. The acceleration of head movements along the x-axis may be indicative of embodiment while in gameplay, as one often moves their head sideways to move away from shots fired by the enemy. Indeed, levels of embodiment have been linked to altered perceptions of time (Charbonneau et al., 2017; Unruh et al., 2021, 2023). In a similar vein, the Up-Down shifts feature, present in Q1, suggests that the players are continuously scanning the scene and engaged. This is expected for fully immersed gamers, as players need to get supplies, which could be on the ground, pick up objects and throw them, and engage in combat with enemies appearing, for example, on floors below you, as shown in Figure 1. In contrast, less immersive screen-based first-person shooter games have shown eye gaze patterns to be mostly in the center of the screen, where the aiming reticle is usually placed (Kenny et al., 2005; El-Nasr and Yan, 2006). SDNN, in turn, characterizes the heart rate variability of the players and has been linked to emotional states (Shi et al., 2017), stress (Wu and Lee, 2009; Pereira et al., 2017), and mental load (Hao et al., 2022), which in turn, have also been shown to modulate the perception of time. This suggests that physiological responses, as indicated by HRV measures, could also significantly influence time perception in VR.

For the top EEG measures, two out of three top measures corresponded to coherence measures in the beta frequency band. The electrodes showing the strongest involvement were located over the pre-frontal cortex areas. The pre-frontal cortex has been linked with temporal processing with activation in the right pre-frontal cortex has been reported during time perception tasks (Üstün et al., 2017). The inter-hemispheric PMSC measure msc-beta-F1-F2 may be quantifying this activation. Moreover, beta-band activation has also been linked with perception of time across multiple studies (e.g., Ghaderi et al., 2018; Damsma et al., 2021). The work by Li and Kim (2021) also showed that beta band activity around the Fpz region could be linked to task complexity, which in turn, was shown to also modulate time perception. In a similar vein, the work by Wiener et al. (2018) showed that transcranial alternating current stimulation over the fronto-central cortex at beta frequency could shift the perception of time to make stimuli seem longer in duration. Such properties could be captured by the PMSC-AM feature msc-beta-mbeta-Fpz-Fp2. Lastly, cortical gamma-theta coupling has been linked to mental workload (Baldauf et al., 2009; Gu et al., 2022), whereas theta-gamma coupling to working memory (Lisman and Jensen, 2013; Park et al., 2013), which itself has been shown to modulate perception of time (Pan and Luo, 2011). As the puzzle-solving tasks often require the use of short-term working memory, this feature is likely quantifying this aspect.

For Q2, four of the top eight features (i.e., IQR-acc-x, SDNN, msc-beta-F1-F2, and msc-beta-mbeta-Fpz-Fp2) overlap with those seen with Q1, suggesting their importance for time perception monitoring and the need for a multimodal system to combine information from EEG, ECG and head movements. The other four provide alternate views of HRV and EEG modulations. For example, pNN50 has been shown to be an HRV correlate of focus (Won et al., 2018). High levels of focus and attention have been shown to be a driving factor for losing sense of time in VR (Winkler et al., 2020). In turn, inter-hemispheric differences in alpha band have also been linked to time perception (Anliker, 1963; Contreras et al., 1985). Lastly, several previous works have linked the theta-beta ratio to attentional control (Putman et al., 2013; Morillas-Romero et al., 2015; Angelidis et al., 2016, 2018). While the ratio is usually computed using frequency bands computed over a certain analysis window, the PMSC-AM feature msc-beta-mtheta-Fp1-Fpz computes the temporal dynamics of this ratio over the window, thus may capture temporal attention changes more reliably. As with focus, high attention levels have been linked to losing sense of time in VR (Winkler et al., 2020).

### 5.3. Monitoring time perception

The high correlation coefficients observed between the predicted and true subjective ratings for Q1 and Q2, as shown in Figures 6A, B, are not only indicative of the accuracy of the proposed feature selection but also highlight the potential of using such features to effectively characterize time perception in VR. The fact that the error-based measures from the chance regressor were almost three times higher than those from the proposed method underscores the importance of careful feature selection and the use of machine learning techniques in predicting time perception. The significant difference in figures-of-merit between the chance regressor and the proposed regressors, as revealed by the Kruskal–Wallis test, further emphasizes the enhanced performance and effectiveness of the present study. This finding is particularly encouraging as it suggests that the proposed method could be used to enhance the design and evaluation of VR experiences by providing a more nuanced understanding of how users perceive time in VR. Moreover, the overlap of top features between Q1 and Q2 suggests that there are common underlying mechanisms in different aspects of time perception in VR, reinforcing the need for a multimodal system that combines information from EEG, EOG, ECG, and head movements.

### 5.4. Recommendations, study limitations, and future work

The experiments described herein have shown the importance of a multimodal system to characterize time perception while immersed in VR. To characterize aspects of time flowing differently, features from four modalities—EEG, ECG, EOG, and accelerometry—were shown to be crucial, thus signaling the importance of an instrumented headset. The aspect of time flowing differently also showed significant correlations with aspects related to immersion, thus suggesting that the developed instrumented headset could provide useful insights for overall monitoring of immersive media quality of experience. If interested in monitoring only aspects of the users losing sense of time while in VR, our results suggest that head movement features and HRV measures can achieve reliable results. Such findings could potentially be achieved with accelerometer data already present in VR headsets and with a heart rate monitor. Additionally, other performance measures such as gameplay duration and score could also provide additional support to the self-assessed ratings of time perception.

The instrumented headset, nonetheless, could provide neural correlates of additional factors related to the overall experience and other aspects of time perception. In fact, one modality that was not explored here was EDA. Electrodermal activity has been used in VR to measure the immersive experience (Egan et al., 2016) as well as player arousal states (Klarkowski et al., 2016). As arousal has been linked to attention and emotional resources, it has also been linked to time perception (Angrilli et al., 1997; Mella et al., 2011). As such, future studies could explore the use of EDA-derived features. Recent innovations in VR headset development are already exploring the inclusion of such sensors directly on the headset (Bernal et al., 2022).

While this study provides promising results, some limitations should be acknowledged. First, as the study was conducted amidst the first COVID-19 lockdown, it has a small number of participants, thus limiting the generalizability of our findings. Moreover, the presence of potential multicollinearity among the predictors in our multiple regression model could affect the interpretability of our findings. Despite these limitations, this study should be considered a feasibility study, exploring the potential of a multimodal system to characterize time perception while immersed in VR. Increasing the sample size in future studies would improve the statistical power and strengthen the validity of our results. Moreover, future studies should consider using a wider range of stimuli to investigate better the relationship between immersion, presence, and time perception in VR, as well as their role in overall quality of experience.

## 6. Conclusions

In this study, we examined time perception in a highly immersive VR environment using a combination of physiological signals, including head movement, heart rate variability, EEG, and EOG measured from sensors embedded directly into the VR headset. Experimental results show that participants experienced a high degree of time distortion when playing the game. Top features were found and used to characterize the gamers' sense of time perception using a simple Gaussian process regressor. An in-depth analysis of these top features were performed. Results showed that the proposed models were able to characterize the gamers' perception of time significantly better than chance. Ultimately, it is hoped that the insights and models shown herein can be used by the community to understand better the relationship between immersion and time perception in virtual environments, thus leading to improve immersive media experiences.

## Data availability statement

The raw data supporting the conclusions of this article will be made available by the authors, without undue reservation.

## Ethics statement

The studies involving human participants were reviewed and approved by INRS Ethics Committee. The patients/participants provided their written informed consent to participate in this study.

## Author contributions

M-AM conducted the experiments, analyzed the data, and wrote the first draft of the paper. All authors conceived and designed the study and the experiment analysis, contributed to manuscript revisions, approved the final version of the manuscript, and agree to be held accountable for the content therein.

## Footnotes

^1^ https://www.mathworks.com/matlabcentral/fileexchange/84692-ecg-class-for-heart-rate-variability-analysis
